# *In silico* assessment of the effects of quinidine, disopyramide and E-4031 on short QT syndrome variant 1 in the human ventricles

**DOI:** 10.1371/journal.pone.0179515

**Published:** 2017-06-20

**Authors:** Cunjin Luo, Kuanquan Wang, Henggui Zhang

**Affiliations:** 1 School of Computer Science and Technology, Harbin Institute of Technology (HIT), Harbin, China; 2 School of Physics and Astronomy, The University of Manchester, Manchester, United Kingdom; 3 Space Institute of Southern China, Shenzhen, China; Georgia State University, UNITED STATES

## Abstract

**Aims:**

Short QT syndrome (SQTS) is an inherited disorder associated with abnormally abbreviated QT intervals and an increased incidence of atrial and ventricular arrhythmias. SQT1 variant (linked to the rapid delayed rectifier potassium channel current, *I*_Kr_) of SQTS, results from an inactivation-attenuated, gain-of-function mutation (N588K) in the *KCNH2*-encoded potassium channels. Pro-arrhythmogenic effects of SQT1 have been well characterized, but less is known about the possible pharmacological antiarrhythmic treatment of SQT1. Therefore, this study aimed to assess the potential effects of E-4031, disopyramide and quinidine on SQT1 using a mathematical model of human ventricular electrophysiology.

**Methods:**

The ten Tusscher *et al*. biophysically detailed model of the human ventricular action potential (AP) was modified to incorporate *I*_Kr_ Markov chain (MC) formulations based on experimental data of the kinetics of the N588K mutation of the *KCNH2*-encoded subunit of the *I*_Kr_ channels. The modified ventricular cell model was then integrated into one-dimensional (1D) strand, 2D regular and realistic tissues with transmural heterogeneities. The channel-blocking effect of the drugs on ion currents in healthy and SQT1 cells was modeled using half-maximal inhibitory concentration (IC_50_) and Hill coefficient (nH) values from literatures. Effects of drugs on cell AP duration (APD), effective refractory period (ERP) and pseudo-ECG traces were calculated. Effects of drugs on the ventricular temporal and spatial vulnerability to re-entrant excitation waves were measured. Re-entry was simulated in both 2D regular and realistic ventricular tissue.

**Results:**

At the single cell level, the drugs E-4031 and disopyramide had hardly noticeable effects on the ventricular cell APD at 90% repolarization (APD_90_), whereas quinidine caused a significant prolongation of APD_90_. Quinidine prolonged and decreased the maximal transmural AP heterogeneity (*δV*); this led to the decreased transmural heterogeneity of APD across the 1D strand. Quinidine caused QT prolongation and a decrease in the T-wave amplitude, and increased ERP and decreased temporal susceptibility of the tissue to the initiation of re-entry and increased the minimum substrate size necessary to prevent re-entry in the 2D regular model, and further terminated re-entrant waves in the 2D realistic model. Quinidine exhibited significantly better therapeutic effects on SQT1 than E-4031 and disopyramide.

**Conclusions:**

The simulated pharmacological actions of quinidine exhibited antiarrhythmic effects on SQT1. This study substantiates a causal link between quinidine and QT interval prolongation in SQT1 and suggests that quinidine may be a potential pharmacological agent for treating SQT1 patients.

## Introduction

Short QT syndrome (SQTS) is a cardiac disorder associated with abnormally abbreviated QT intervals and an increased incidence of atrial and ventricular arrhythmias or sudden cardiac death (SCD) [1–4]. Short QT interval in SQTS is commonly less than 360 ms with a range of 220 to 360 ms; another important feature in SQTS is the tall, symmetrical or asymmetrical peaked T wave morphology. The term ‘short QT interval’ was first described by Gussak *et al*. [1] in 2000, and it was recognized as the distinct clinical entity. The familial nature and characteristics of this sudden death syndrome were simply demonstrated by Gaita *et al*. [2] in 2003. These reports led to the recognition of SQTS as a new, distinct disease and have been quickly followed by additional human case reports over the past decade. The molecular basis for SQTS associated with various mutations in 6 different genes: *KCNH2* [5], *KCNQ1* [6], *KCNJ2* [7], *CACNA1C* [8], *CACNB2b* [8] and *CACNA2D1* [9], have been reported. The corresponding short QT syndrome has been termed from SQT1 to SQT6 depending on the chronological order of discovery. Among these dominant mutations, either a gain-in-function of the potassium channel (linked to *KCNH2*, *KCNQ1* and *KCNJ2* gene) or a loss-in-function of the calcium channel (linked to *CACNA1C*, *CACNB2b* and *CACNA2D1* gene) across the membrane of cardiac muscle cells has been observed.

*KCNH2*-linked short QT syndrome (SQT1) was first identified in a 51-year-old male presenting a corrected QT interval (QTc) of 288 ms. Based on genetic analysis of *KCNH2*, a single amino-acid residue substitution (asparagine-to-lysine, N588K) in the turret (also called S5-pore linker) region of the human ether-à-go-go-related gene (*hERG*) potassium channels was discovered. Results from *in vitro* standard whole-cell patch clamp recordings have revealed that this mutation leads to an increased activity of rapid delayed rectifier potassium current (*I*_Kr_) in the *hERG* potassium channels. The N588K mutation leads to loss of normal rectification of *I*_Kr_ over the physiological range of membrane potentials, resulting in a substantial gain of function during phase 2 and 3 of the cardiac action potential (AP), and thereby accelerated ventricular repolarization [5,10,11]. In the previous studies, Itoh *et al*. [12] and Adeniran *et al*. [13] investigated the mechanisms how *KCNH2* gene-mediated channel defects cause life-threatening arrhythmias in SQT1, and have in detailed shown that SQT1 abbreviated the AP duration (APD) and effective refractory period (ERP) and reduced the maximal slopes of APD restitution (APD-R) and ERP restitution (ERP-R) curves, which was consistent with the poor rate-dependent of QT intervals and consequently shortened the QT interval and raised the T-wave amplitude in SQT1 patients.

Due to the malignant ventricular arrhythmia in SQTS, the implantable cardioverter defibrillator (ICD) is primarily recommended for patients with a diagnosis of SQTS. Despite the success of this technique, a problem with ICD in SQTS is an increased risk of an inappropriate shock from the ICD. Moreover, the QT interval does not fall within the normal range over time by using the ICD. SQTS patients may benefit from subcutaneous ICD therapy, but additional clinical cases are needed to affirm the efficacy and safety of such device [14]. Although the ICD remains the mainstay of treatment for SQTS patients, pharmacological therapy may be useful as an adjunct to the ICD therapy or may restore the normal QT interval and suppress atrial and ventricular fibrillation. However, to date, available data regarding pharmacological treatment for SQTS patients are very limited. An *in vivo* study by Gaita *et al*. [15] tested four antiarrhythmic drugs including hydroquinidine, flecainide, ibutilide and sotalo in SQT1 patients, to determine whether they could prolong the QT interval into the normal range. They reported quinidine prolonged the QT interval and ERP and effectively prevented ventricular arrhythmias in SQT1 patients [15]. Quinidine also restored the QT interval toward the normal range [16]. In a one-year follow-up, the SQT1 patients who treat with hydroquinidine remained asymptomatic, and no further episode of arrhythmias was detected. An *in vitro* study by McPate *et al*. [17] evaluated the impact of selected class I and class III five antiarrhythmic drugs including E-4031, disopyramide, amiodarone, quinidine and propafenone on SQT1 N588K mutant channels.

In summary, pharmacological treatment for SQTS patients has not yet been established. Quinidine seems to be a first-line therapeutic option in patients with SQTS. However, little is known about the mechanisms of actions of quinidine on SQT1. Moreover, drugs exert effects spanning multiscale levels from ion channel to tissue, organ and whole body, making investigation using experimental technique tough and rather expensive [18]. *In silico* models of the heart provide a powerful means of investigating some questions that do not lend themselves readily to *in vitro* or *in vivo* studies. Elucidating pharmacological effects of drugs on SQTS falls into this category as it is not easy to conceive of biological experiments that would accurately assess the effects of drugs on the known SQTS. Furthermore, several recent reviews [19–23] have highlighted the power of *in silico* models to investigate arrhythmias and predict pharmacological effects of drugs. Recent studies [18,24–26] have explicitly shown that *in silico* models have proven to be useful in predicting the effects of drugs on arrhythmias. Therefore, this study was undertaken to assess the effects of quinidine on SQT1 by using computational human ventricular cell and tissue models, and consequent effects on QT interval prolongation and prevention and termination of re-entrant ventricular arrhythmias in this variant of SQTS.

## Materials and methods

### Model development

**Fig 1:**
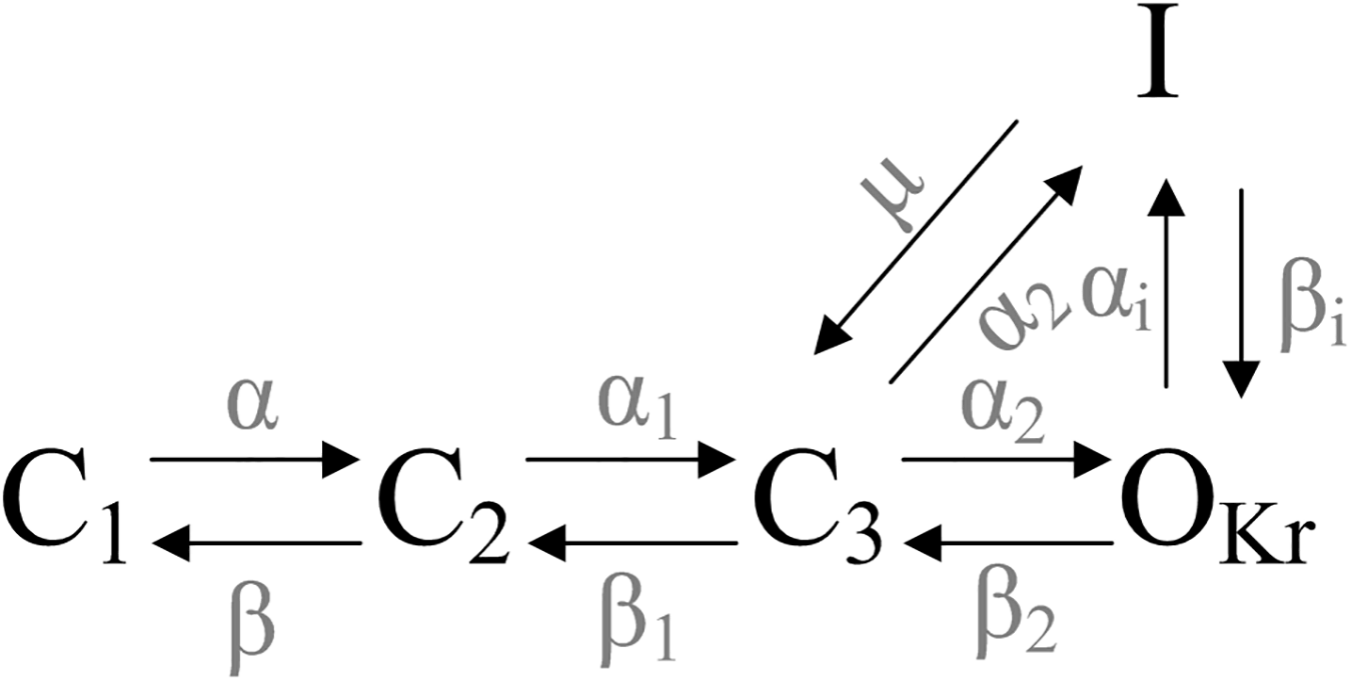
A schematic for the *I*_Kr_ MC model used in this study. *O*_Kr_ is the open state. *C*_1_-*C*_3_ are the closed states. *I* is the inactivated state.

The ten Tusscher *et al*. [27] biophysically detailed computer model for human endocardial (ENDO), mid-myocardial (MIDDLE) and epicardial (EPI) ventricular APs was used in this study. *I*_Kr_ dynamic equations in the original model were replaced with a previously-described Markov chain (MC) formulation [13] of *hERG* wild-type (WT) and N588K *I*_Kr_ channel. These properties include that (i) a shift in the voltage-dependence of mutant *I*_Kr_ channel, and (ii) an increased *I*_Kr_ during the repolarization phase in the AP [5,11,17,28]. A schematic for the *I*_Kr_ MC model is shown in Fig 1. The *I*_Kr_ MC model made up of an open state (*O*_Kr_), three closed states (*C*_*1*_, *C*_*2*_ and *C*_*3*_), and an inactivated state (*I*), which is described as:

$$I_{Kr}=G_{Kr}\times O_{Kr}\times (V-E_{Kr})$$ (1)

$$G_{Kr}=0.0243\times {\left[K^{+}\right]}_{o}^{0.59}$$ (2)

$$E_{Kr}=\frac{RT}{F}\log\frac{{\left[K^{+}\right]}_{o}}{{\left[K^{+}\right]}_{i}}$$ (3)

$$C_{1}=\beta \cdot C_{2}-\alpha \cdot C_{1}$$ (4)

$$C_{2}=\alpha \cdot C_{1}-\beta \cdot C_{2}+\beta_{1}\cdot C_{3}-\alpha_{1}\cdot C_{2}$$ (5)

$$C_{3}=\alpha_{1}\cdot C_{2}-\beta_{1}\cdot C_{3}-2\cdot \alpha_{2}\cdot C_{3}+\mu \cdot I+\beta_{2}\cdot O_{Kr}$$ (6)

$$I=\alpha_{2}\cdot C_{3}-\mu \cdot I+\alpha_{i}\cdot O_{Kr}-\beta_{i}\cdot I$$ (7)

$$O_{Kr}=1-(C_{1}+C_{2}+C_{3}+I)$$ (8)

where *G*_Kr_ is the maximal conductance, *O*_Kr_ is the open probability, [*K*^+^]_o_ and [*K*^+^]_i_ are extracellular and intracellular *K*^+^ concentration, respectively. *V* is the transmembrane potential and *E*_Kr_ is the *K*^+^ equilibrium (or reversal) potential. Modified *I*_Kr_ formulations for WT and N588K conditions are shown in the Supporting Information.

A simple pore block theory [29] was used to model drug/ion channel binding interactions. The maximum conductance of cardiac specific ion channels was reduced according to the Hill equation. The respective reduction of ion currents in the presence of E-4031, disopyramide or quinidine was determined by using half maximal inhibitory concentration (IC_50_) and Hill coefficient (nH) values taken from literatures. The blocking potency of these drugs on ionic currents is shown in Table 1, Table 2 and Table 3, respectively. E-4031 is a pure *I*_Kr_ blocker [30] and it does not affect the *Na*^+^ and *Ca*^2+^ inward currents [31], whereas disopyramide and quinidine are multiple current blockers [15,32]. The therapeutic concentration of E-4031 is 3~4 nM [33], disopyramide is 8.3~13.0 μM (corresponding to be 2.8~4.4 mg/L) [34], and quinidine is 3.8~10.2 μM [35]. Several doses (3.0, 3.5 and 4.0 nM for E-4031, 9, 10 and 13 μM for disopyramide, and 4, 7 and 10 μM for quinidine) were selected and simulated to compare the effects on SQT1 related N588K mutation in *KCNH2*.

**Table 1:** Cardiac ion currents and conductivities (% of original value) in the presence of 3.0, 3.5 and 4.0 nM E-4031.

Current	IC50	*nH*	Conductivity	Source
**WT** ***I***_**Kr**_	15.96±0.04 nM	0.74±0.05	86.0/83.2/80.0%	[17,38]
**N588K *I***_**Kr**_	183±0.04 nM	1.07±0.04	99.0/98.8/98.0%	

**Table 2:** Cardiac ion currents and conductivities (% of original value) in the presence of 9, 10 and 13 μM disopyramide.

Current	IC50	*nH*	Conductivity	Source
**WT** ***I***_**Kr**_	10.66±0.04 nM	1.07±0.05	60.0/51.7/39.1%	[17,38]
**WT *I***_**Na**_	168.4 μM	1.09	96.7/95.6/95.0%	[39]
**WT *I***_**to**_	259 μM	1.07	97.6/97.0/94.3%	[32]
**WT *I***_**CaL**_	1036.7 μM	1.0	99.3/99.0/98.7%	[39]
**N588K *I***_**Kr**_	15.77±0.04 μM	0.63±0.04	63.9/57.1/49.1%	[17,38]
**N588K *I***_**Na**_	168.4 μM	1.09	96.7/95.6/95.0%	[39]
**N588K *I***_**to**_	259 μM	1.07	97.6/97.0/94.3%	[32]
**N588K *I***_**CaL**_	1036.7 μM	1.0	99.3/99.0/98.7%	[39]

**Table 3:** Cardiac ion currents and conductivities (% of original value) in the presence of 4, 7 and 10 μM quinidine.

Current	IC50	*nH*	Conductivity	Source
**WT** ***I***_**Kr**_	0.62±0.03 μM	0.93±0.06	17.0/10.0/7.0%	[17,38]
**WT *I***_**Ks**_	Not given	Not given	75.0/60.0/45.2%	[40]
**WT *I***_**to**_	3.9 μM	1.0	46.5/38.9/25.3%	[41]
**WT *I***_**Na**_	0.17 nM	1.0	79.6/77.0/59.3%	[42]
**WT *I***_**CaL**_	14.9±1.5 μM	1.1±0.1	83.2/72.9/61.2%	[43]
**WT *I***_**NaCa**_	Not given	Not given	95.0/90.0/86.5%	[43]
**WT *I***_**NaL**_	12.0±0.7 μM	1.0	28.3/37.7/52.3%	[35]
**N588K** ***I***_**Kr**_	2.16±0.02 μM	0.92±0.04	44.0/29.8/19.1%	[17,38]
**N588K *I***_**Ks**_	Not given	Not given	75.0/60.0/45.2%	[40]
**N588K *I***_**to**_	3.9 μM	1.0	465.6/38.9/25.3%	[41]
**N588K *I***_**Na**_	0.17 nM	1.0	79.6/77.0/59.3%	[42]
**N588K *I***_**CaL**_	14.9±1.5 μM	1.1±0.1	83.2/72.9/61.2%	[43]
**N588K *I***_**NaCa**_	Not given	Not given	95.0/90.0/86.5%	[43]
**N588K *I***_**NaL**_	12.0±0.7 μM	1.0	28.3/37.7/52.3%	[35]

Effects of a combined action of blocking of multiple ion currents in the presence of quinidine at 4, 7 and 10 μM doses together with theoretical (75.0 and 60.0%) and experimental (45.2%) blocking of *I*_Ks_ and with theoretical (95.0 and 90.0%) and experimental (86.5%) blocking of *I*_NaCa_.

### Single cell model and AP simulations

The validated *I*_Kr_ MC model was then incorporated into the ten Tusscher *et al*. [27] cell model. Specifically, the single cell model can be modeled by using the following ordinary differential equation (ODE):

$$\frac{dV}{dt}=-\frac{I_{ion}+I_{stim}}{C_{m}}$$ (9)

where *t* is time, *C*_m_ is the cell membrane capacitance per unit surface area, *I*_stim_ is the external stimulus current and *I*_ion_ is the sum of all transmembrane ionic currents. Particularly, the component of the late sodium current (*I*_NaL_) from the ORd dynamic model [36] was incorporated. The model code used in this study was downloaded from http://www-binf.bio.uu.nl/khwjtuss/. The cell model was paced with an amplitude of -52 pA/pF for 1 ms and a basic cycle length (BCL) of 800 ms, which roughly corresponds to 75 bpm of the normal human heart. After the pacing, the resulting ventricular cell APD at 90% repolarization (APD_90_) of the WT and N588K ENDO, MIDDLE and EPI cells were measured. APs were elicited with an S1-S2 protocol consisting of 20 S1 stimuli and an S2 stimulus. The S1 was applied at a frequency of 1.25 Hz and -52 pA/pF strength for 1 ms. The S2 was applied at varying diastolic intervals (DI) after the AP-evoked by the last S1. The APD-R curve was generated by decreasing the DIs and plotting the APD_90_ evoked by the S2 against the DIs. The ERP was measured as the smallest DI for which the overshoot of the AP- evoked by the S2 reached 80% of the AP-evoked by the 20^th^ S1 at each pacing cycle length (PCL) [37]. The ERP-R curve was generated by plotting the measured EPR against PCLs. Eq 9 was integrated by using the forward Euler method with a time step of 0.02 ms.

### Tissue simulations

Electrical conduction in the multicellular tissue can be described by the following partial differential equation (PDE):

$$C_{m}\frac{\partial V}{\partial t}=-(I_{ion}+I_{stim})+\nabla \cdot (D\nabla V),$$ (10)

where ∇ is the spatial gradient operator, *D* is the diffusion coefficient between ventricular cells.

**Fig 2:**
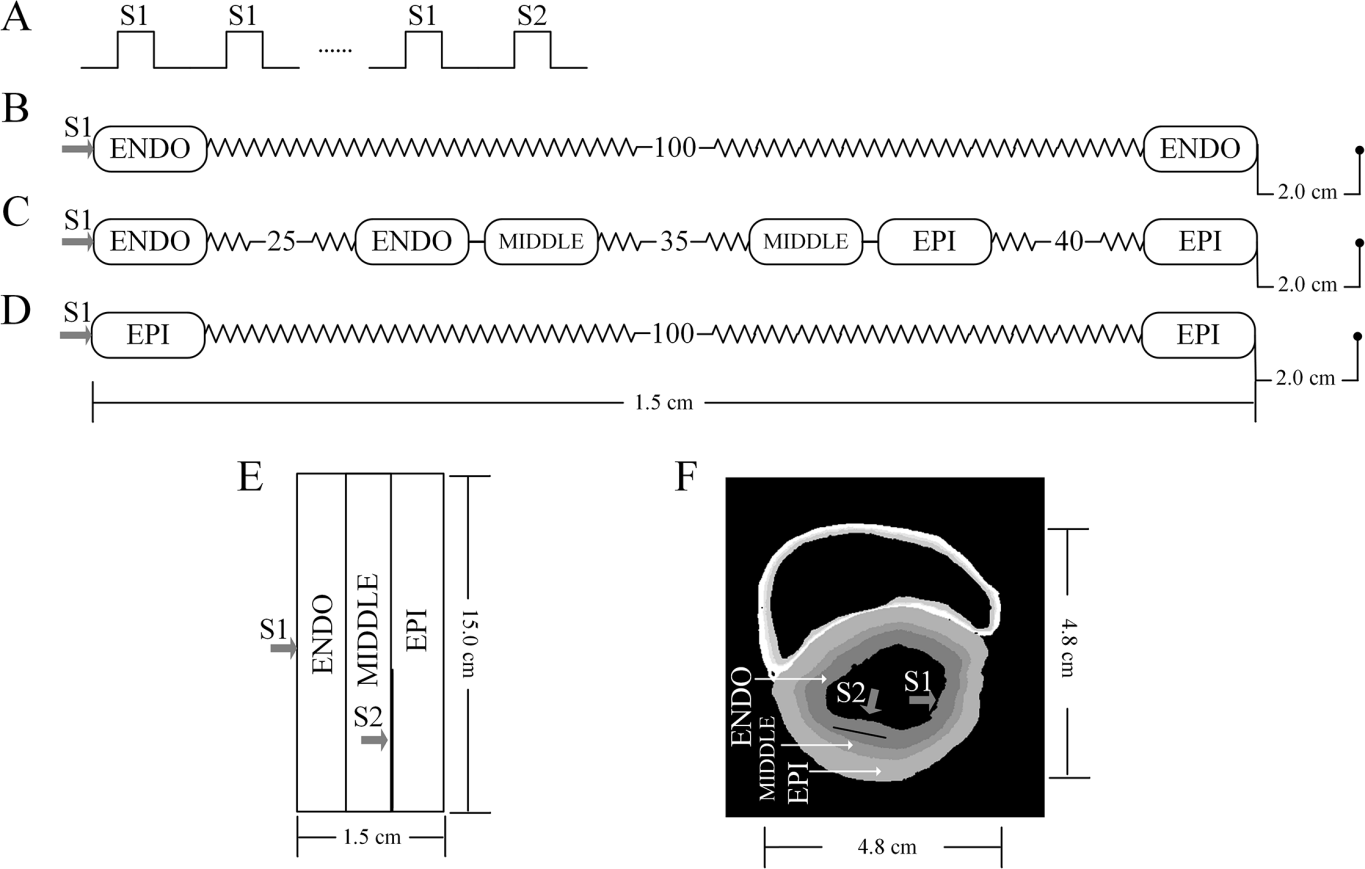
Schematic representation of the 1D strand and 2D regular and realistic models. (A) S1-S2 stimulus protocol. (B) ENDO-to-ENDO connection of a 1D strand model. (C) 1D transmural ventricular strand model. The virtual electrode was placed at a position 2.0 cm away from the EPI end of the strand. (D) EPI-to-EPI connection of a 1D strand model. (E) Schematic representation of the 2D regular model. (F) 2D realistic model.

For one-dimensional (1D) computations, the simulated 1D strand (Fig 2) was 15 mm and employed a spatial resolution of 0.15 mm, close to the ventricular cell length of 80–150 μm, which generated 25 nodes for ENDO (25%), 35 nodes for MIDDLE (35%) and 40 nodes for EPI (40%) cells. Drouin *et al*. [44] reported a 10 to 12 mm thickness of transmural slices. Yan *et al*. [45] reported an average thickness of the wedge preparations of 12.9 ± 1.5 mm. The total length of the 1D strand shows close agreement with these experimental data. The lengths of each sub-region were 3.75 mm, 5.25 mm and 6 mm. The total strand length and proportion for each sub-region are identical to those used in other studies [13,37,46,47] and reliably reproduced a positive T wave in the simulated pseudo-ECG in the control condition. The stimulation protocol (amplitude: -52 pA/pF, duration: 1 ms) was shown in Fig 2A. We constructed ENDO-ENDO (Fig 2B), ENDO-MIDDLE-EPI (Fig 2C) and EPI-EPI (Fig 2D) connection in the 1D cable models. *D* was set at 0.0008 cm^2^/ms, which promoted an electrical excitation conduction velocity (CV) of 52 cm/s through the 1D strand, which is close to the experimental CV of ~50 cm/s [48,49]. *D* was homogenous except for a 5-fold decrease at the MIDDLE-EPI junction, as previously suggested by Gima and Rudy [50].

A pseudo-ECG was calculated as an integral of the transmural gradient of the cell APs at all positions on the strand by using the following expression [50]:

$$\phi_{e}(x\prime )=\frac{\alpha^{2}}{4}\int \left(-\nabla V_{m}\right)\cdot [\frac{1}{r}]dx,$$ (11)

where *α* is the radius of the 1D simulated strand, *dx* is the spatial resolution, *r* is the Euclidean distance from a strand point *x* to the electrode point *x′*. In this study, we placed the virtual electrode at a position 2.0 cm away from the EPI end of the strand. The QT interval was calculated as the time interval determined by definition of the Q-wave onset (*Q*_onset_ = 0.0 mV, *t* = 0 ms) and the point corresponding to the T-wave end (*T*_end_). The time, at which the ECG data fell below a threshold (*V*_thresh_ = 0.01 mV) was defined as *T*_end_.

A regular 2D simulated transmural tissue was modeled by expanding the 1D strand (length of 15 mm) with a width of 150 mm (Fig 2E). The spatial resolution in both length- and width- directions was the same as used in the 1D strand model. In the regular model, re-entrant excitation wave was initiated by an S1-S2 protocol. An S1 stimulus (amplitude: -52 pA/pF, duration: 1 ms) was applied to the ENDO end to evoke a planar excitation wave propagating through MIDDLE towards the EPI end. During the vulnerable window (vulnerable window, during which stimulation produces a solitary wave that propagates in either the anterograde or retrograde direction, is the time window which results in unidirectional block that allows re-entry), an S2 stimulus with the same amplitude and duration as the S1 was applied to the MIDDLE-EPI junction area to evoke unidirectional wave propagation. This action led to the generation of a re-entry that then progressively rotated through the tissue. However, the S2 had variable spatial sizes. Sufficient S2 size is required to provide an adequate re-entrant pathway, which is dependent on the wavelength (wavelength = ERP×CV) of excitation waves. In order to evaluate the critical size of the re-entrant pathway, we estimated the minimum length of S2. This length is an index which indicates the susceptibility of the ventricular tissue to re-entry, i.e., the larger the minimum length, the more difficult the initiation of re-entrant excitation waves. The dominant frequency (DF) of the AP profile from a special site was computed from a time interval between two neighbor AP amplitudes, whose reciprocal provides a measure of the DF. For such an initiated wave, effects of E-4031, disopyramide and quinidine on dynamical behaviors were evaluated in the SQT1 condition. In the 2D realistic model (including 11343 ENDO cells, 6565 MIDDLE cells and 16229 EPI cells), the S1-S2 protocol was applied in the ENDO of the left ventricle (Fig 2F). The S2-evoked excitation wave propagates unidirectionally, leading to the formation of re-entrant excitation wave within the transmural wall.

## Results

### Effects of E-4031, disopyramide and quinidine on SQT1 in the cell model

**Fig 3:**
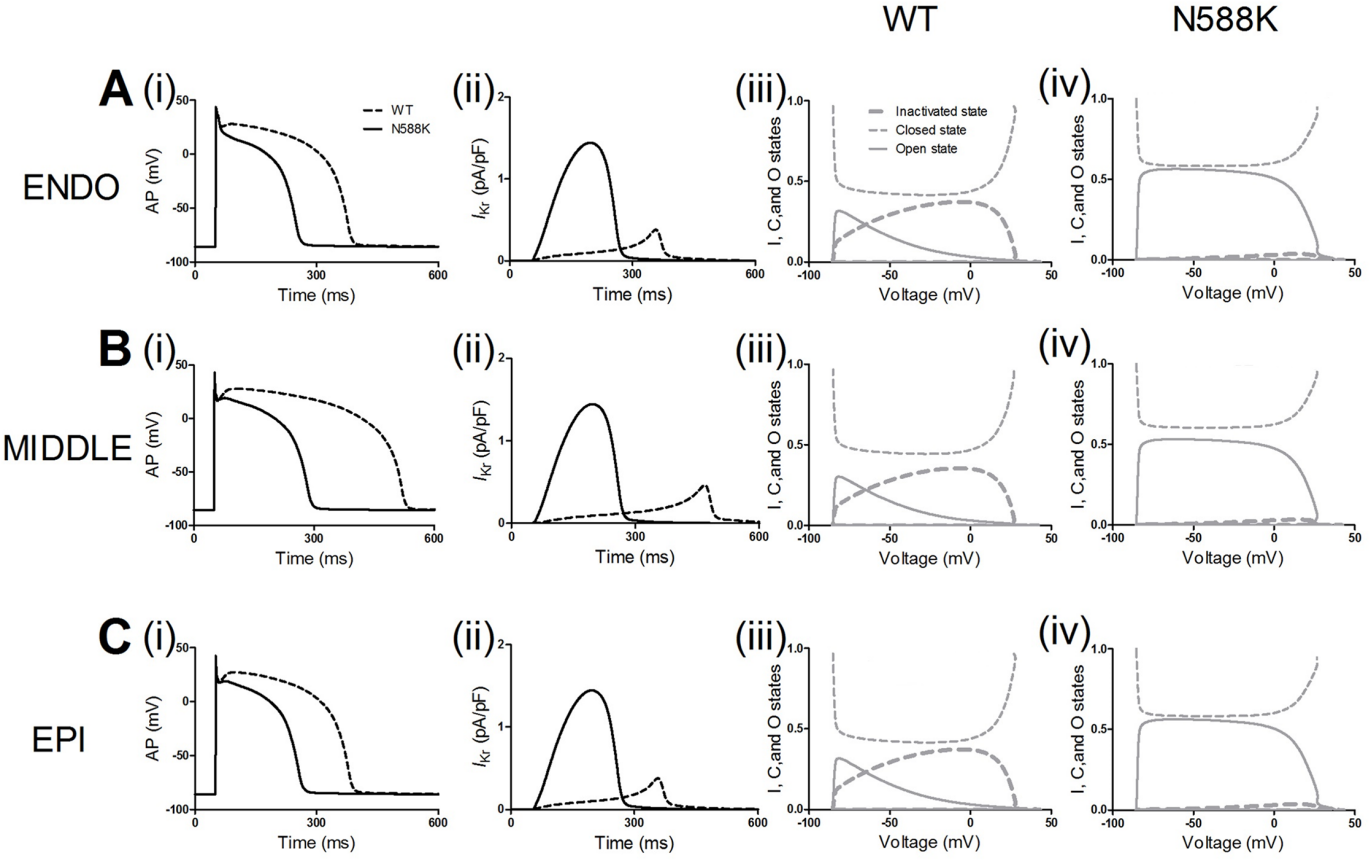
Simulation of APs and *I*_Kr_ time course and channel states. (A) ENDO simulation of steady state APs (i), corresponding *I*_Kr_ current profiles (ii), inactivation, open and closed states of the *I*_Kr_ channels in WT (iii) and N588K (iv) conditions using the *I*_Kr_ Markov chain model. Solid black lines represent WT and dashed black lines represent N588K condition. Solid gray lines represent the open state, dashed gray lines represent inactivated state, and thick dashed gray lines represent the closed state. (B) MIDDLE simulation of steady state APs (i), corresponding *I*_Kr_ current profiles (ii), inactivation, open and closed states of the *I*_Kr_ channels in WT (iii) and N588K (iv) conditions. (C) EPI simulation of steady state APs (i), corresponding *I*_Kr_ current profiles (ii), inactivation, open and closed states of the *I*_Kr_ channels in WT (iii) and N588K (iv) conditions.

**Fig 4:**
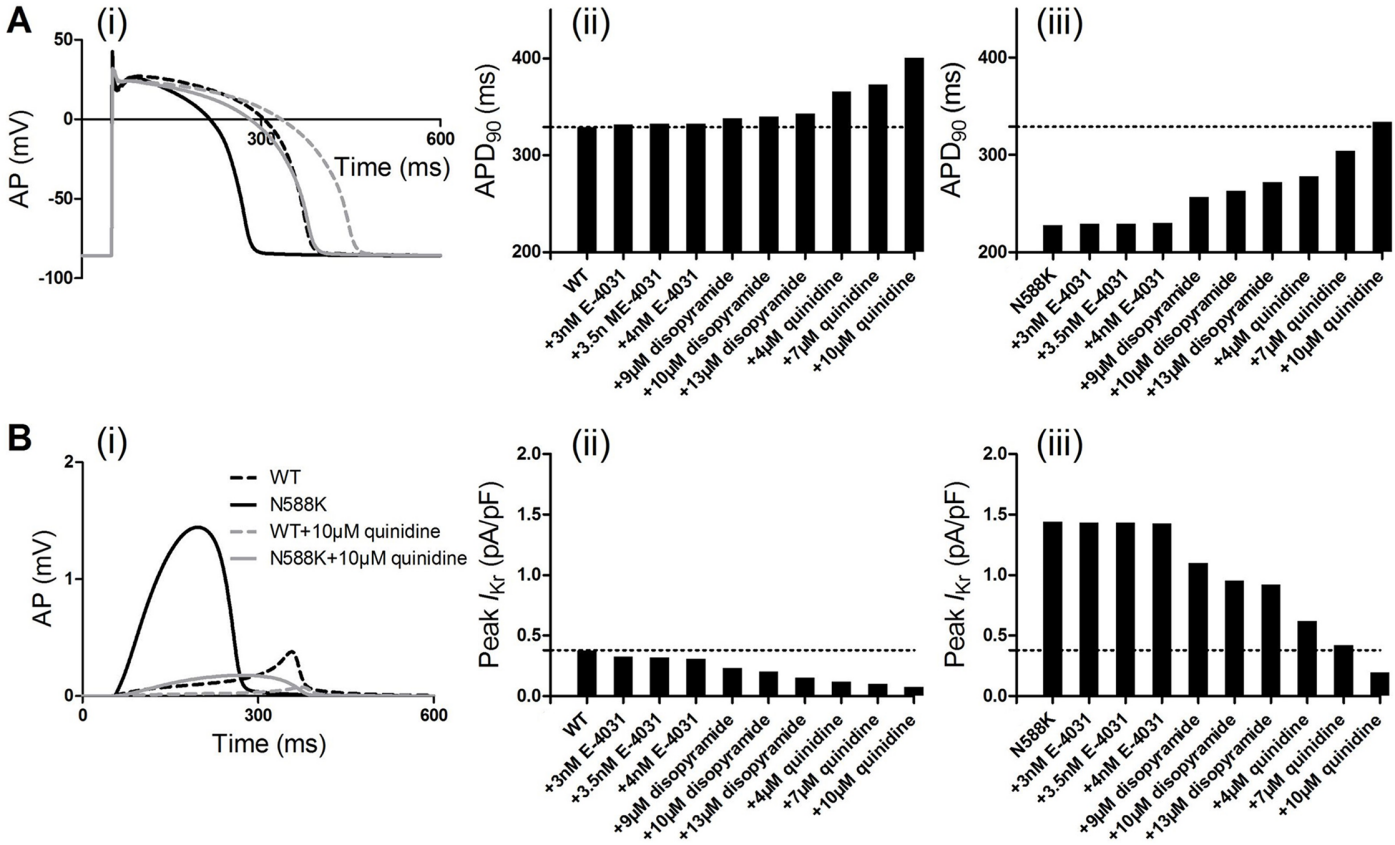
Effects of E-4031, disopyramide and quinidine on human ventricular EPI cells. (A) AP waveforms (i) and corresponding APD_90_ histogram in WT (ii) and N588K (iii) conditions. (B) *I*_Kr_ current profile (i) and peak density in WT (ii) and N588K (iii) conditions. Dashed black lines represent WT condition and solid black lines represent N588K condition. Dashed gray lines represent WT+10μM quinidine condition and solid gray lines represent N588K+10μM quinidine condition.

Fig 3 shows the simulation of APs and *I*_Kr_ profile and channel states in the ENDO (Fig 3A), MIDDLE (Fig 3B) and EPI (Fig 3C) cell model with the MC *I*_Kr_ in the WT and N588K conditions. Fig 4 exhibits the time course of the EPI cell AP waveforms, APD_90_ (Fig 4A) and *I*_Kr_ current profile/peak density (Fig 4B) in the E-4031-, disopyramide- and quinidine-in-action conditions. AP waveforms show the cardiac myocyte transmembrane potential *V* (mV) versus time *t* (ms). The results indicate the following: (i) the WT *I*_Kr_ increased during the time course of the upstroke and plateau phases of the AP, reaching the peak amplitude before a rapid decrease during the final rapid repolarization phase; (ii) The N588K mutant *I*_Kr_ increased more rapidly following the upstroke phase and reached a higher amplitude, leading to APD-shortening effect; (iii) The simulated E-4031 and disopyramide had hardly noticeable effects on APD, whereas quinidine caused a prolongation of APD both in WT and N588K conditions; quinidine at a dose of 10 μM produced a significant prolongation of the APD in the N588K condition, bring it much closer to that in the WT drug-free condition; (iv) Simulated drugs markedly affected the AP peak amplitude but did not affect the resting potential (RP). The effects are due to the reduction of channel conductance, which depends on the actions of drugs that decelerate the repolarization process.

### Effects of E-4031, disopyramide and quinidine on SQT1 in the 1D strand model

**Fig 5:**
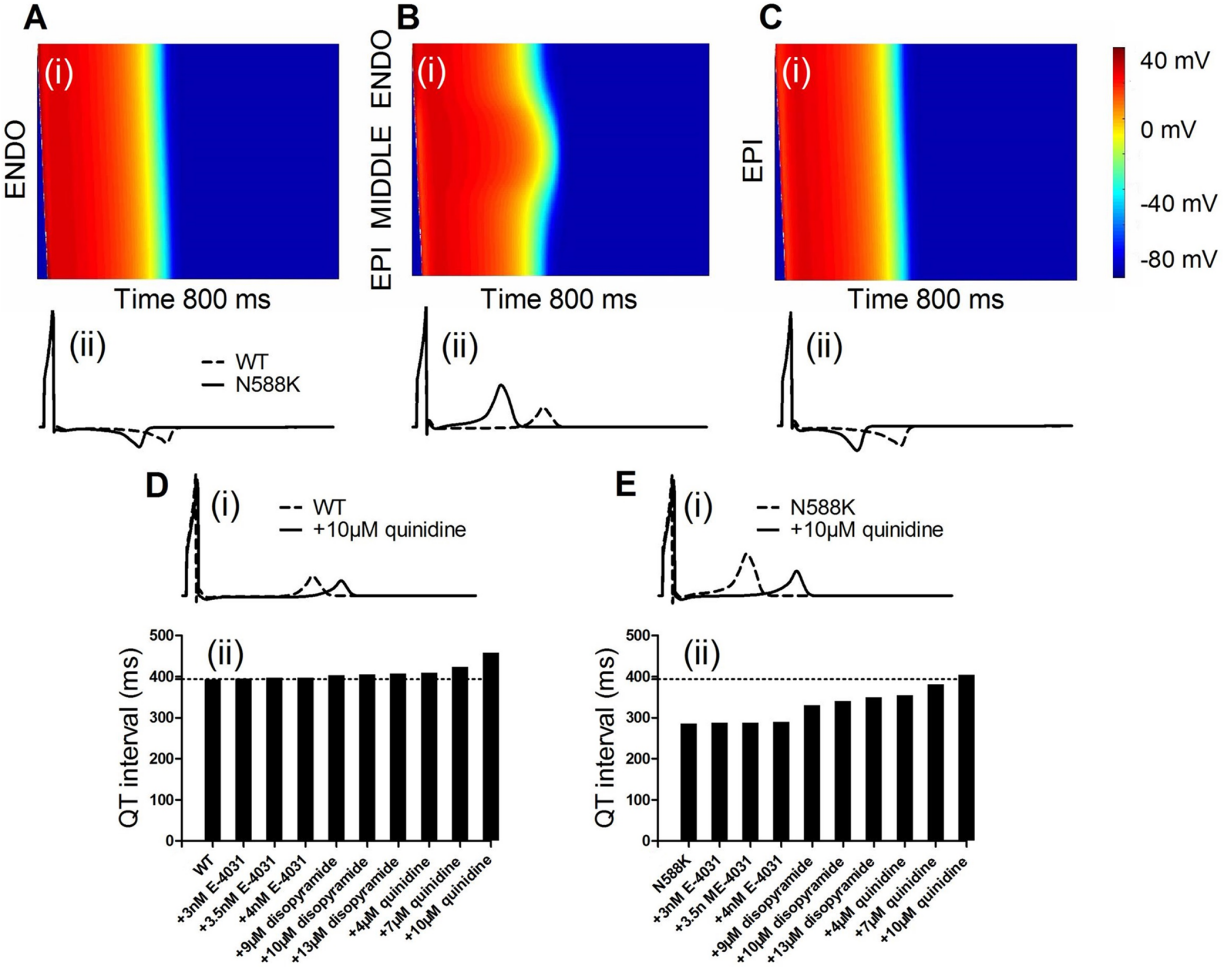
Space-time plot of excitation prolongation across a 1D strand model, the computed pseudo-ECGs and QT intervals. (A) Excitation prolongation across an ENDO-ENDO connection 1D strand model (i) and pseudo-ECGs (ii). Color mapping of membrane voltage along the 1D strand from blue to red. Space runs from top (ENDO) to bottom (EPI). Time runs from left (0 ms) to right (800 ms). The corresponding pseudo-ECGs derived from the propagating electrical excitation wave above. (B) Excitation prolongation across a 1D transmural ventricular strand model (i) and pseudo-ECGs (ii). (C) Excitation prolongation across an EPI-EPI connection 1D strand model (i) and pseudo-ECGs (ii). (D) Superimposed pseudo-ECGs (i) and corresponding QT interval (ii) in WT and drug-in-action conditions by using a 1D transmural ventricular strand model. (E) Superimposed pseudo-ECGs (i) and corresponding QT interval (ii) in N588K and drug-in-action conditions by using a 1D transmural ventricular strand model.

We then simulated the effects of E-4031, disopyramide and quinidine on the electrical activity of the ventricle and the pseudo-ECG traces using the 1D strand model (including ENDO-to-ENDO connection, transmural and EPI-to-EPI connection forms described in the Methods section) at a stimulation frequency of 1.25 Hz. The results are presented in Fig 5. Delivery of a series of super-threshold stimuli (-52 pA/pF) to one end initiated electrical propagation towards the other end of the 1D strand model. From this electrical excitation wave, pseudo-ECG traces were simulated. However, in our initial simulations, the 1D strand model failed to reproduce an unusual increase in the T-wave amplitude recorded from SQT1 patients. To solve this discrepancy problem, an *I*_Kr_ ratio of 1.6:1:1 in the EPI, MIDDLE and ENDO cell model was applied as observed in the experimental study [51]. This experimental study described that *hERG* mRNA expression was ~1.6 times more abundant in the EPI cells than in the MIDDLE cells, consistent with the possible transmural heterogeneity of *I*_Kr_ density. Ten Tusscher *et al*. [27] assumed a uniform *I*_Kr_ density in the ENDO, MIDDLE and EPI cell model. However, when *I*_Kr_ density in the EPI cell was set to be 1.5–1.7 time greater than that in the ENDO and MIDDLE cell, the 1D strand model was then able to generate a higher T-wave amplitude on the computed pseudo-ECG (S1 Fig in the Supporting Information). In Fig 5A(ii) and 5C(ii), both ENDO-to-ENDO connection and EPI-to-EPI connection 1D models failed to reproduce the major feature of SQTS ECG: a significant increase in the positive T wave amplitude in the pseudo-ECG. In this study, we used the 1D transmural strand model (Fig 5B(ii)) with *I*_Kr_ ratio of 1.6:1:1 in the EPI, MIDDLE and ENDO cells to assess the drugs on SQT1.

Compared with the WT condition, the N588K mutation did not affect the depolarization phase but accelerated the repolarization process of the ventricular tissue. Simulated E-4031, disopyramide and quinidine delayed the repolarization phase in the both WT and N588K conditions. The actions of the drugs caused QT prolongation and lower amplitude in the T-wave morphology (Fig 5D(i) and 5E(i)). Then, we calculated QT intervals extracted from the simulation data as showed in Fig 5D(ii) and 5E(ii) in WT and N588K conditions, respectively. The pseudo-ECG traces show an increment in the QT interval in the presence of 3, 3.5 and 4 nM E-4031, 9, 10 and 13 μM disopyramide, and 4, 7 and 10 μM quinidine, which changed from 394 (control) in the WT condition to 396, 398, 398, 404, 406, 408, 410, 424 and 458 ms, respectively. For the same condition, the QT interval was prolonged from 286 in the N588K condition to 288, 288, 290, 330, 340, 350, 355, 382 and 405 ms, respectively. The N588K mutant QT intervals in the presence of 7 and 10 μM quinidine are 382 and 405 ms, which are within the physiological range of QT interval between 363 and 421 ms reported for 1 Hz stimulation frequency, with a little difference of the stimulation frequency of 1.25 Hz in the present computer simulations.

**Fig 6:**
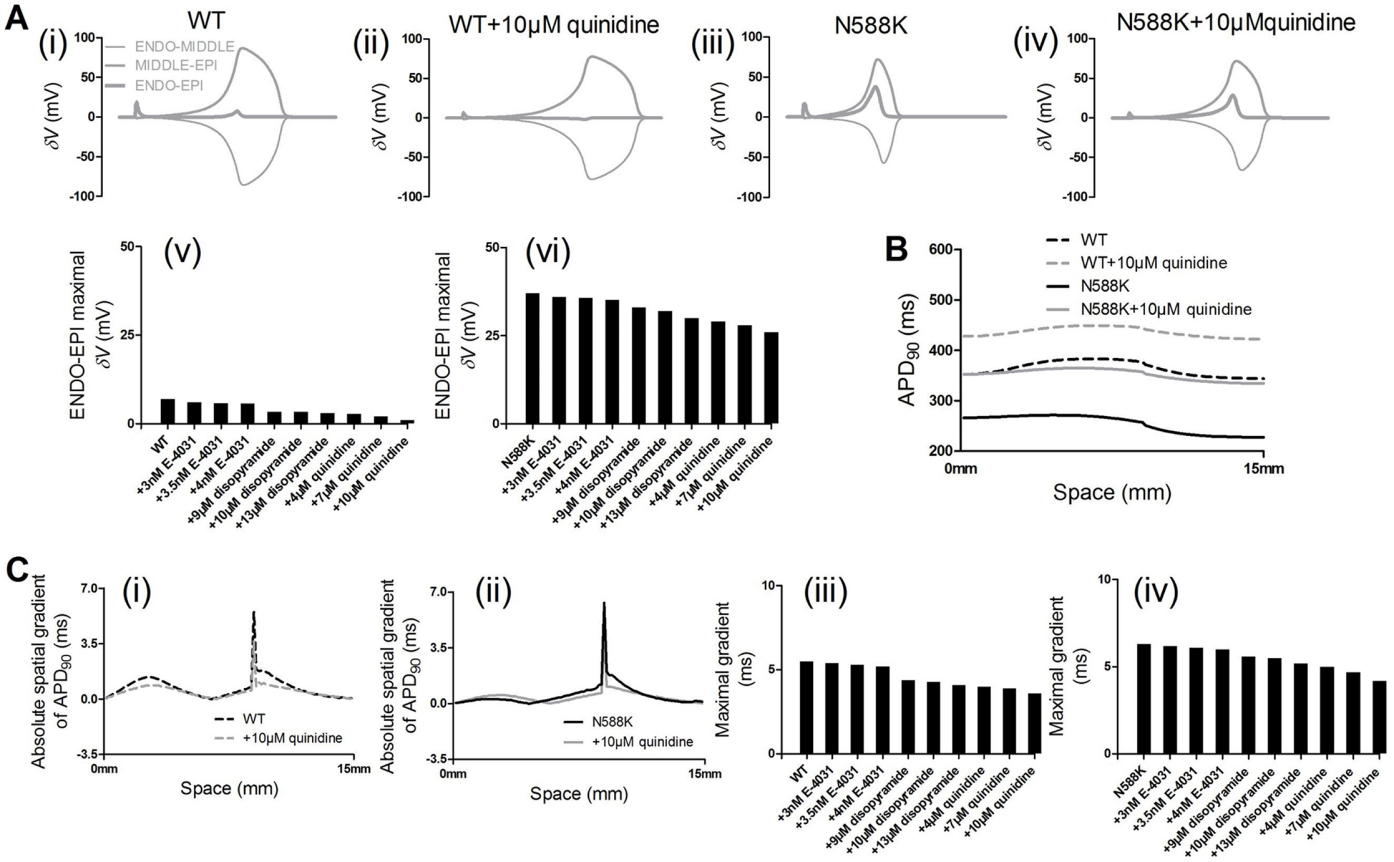
Action potential heterogeneity (*δV*) between single ENDO, MIDDLE and EPI cells, transmural APD_90_ distribution and its spatial gradient along a 1D transmural strand model. (A) Plots of *δV* against time for WT (i), WT+10μM quinidine (ii), N588K (iii) and N588K+10μM quinidine (iv) conditions, and maximal *δV* during repolarization process between ENDO-EPI cells in the WT (v) and N588K (vi) conditions. (B) Spatial distribution of APD_90_ across the 1D transmural strand in the WT, N588K and quinidine-in-action conditions. Dashed black lines for WT condition and solid black lines for N588K condition, dashed gray lines for WT+10μM quinidine condition and solid gray lines for N588K+10μM quinidine condition. (C) The spatial gradient of APD_90_ across the 1D transmural strand in the WT (i), N588K (ii) and quinidine-in-action conditions, and maximal spatial gradient of APD_90_ in the WT (iii) and N588K (iv) conditions.

To examine the factors responsible for the significantly decreased T-wave amplitude, we analyzed the effects of E-4031, disopyramide and quinidine on the AP heterogeneity (*δV*), as well as the transmural dispersion of APD across the 1D strand tissue. The results are presented in Fig 6. Differences of *δV* between EPI, MIDDLE and ENDO cells are shown in Fig 6A. Compared with the WT condition, the N588K mutation increased the *δV*, which contributed to the increased T-wave amplitude on the pseudo-ECG. With the presence of quinidine both in WT and N588K conditions, the *δV* was significantly decreased, which subsequently caused the decreased T-wave amplitude. Spatial distribution of APD_90_ across the 1D transmural strand model was shown in Fig 6B. With a ratio of 1.6:1:1 for the EPI *I*_Kr_ density to MIDDLE and ENDO *I*_Kr_ density, the N588K mutation augmented dispersion of APD_90_ across the 1D strand model. Quinidine decreased the dispersion (Fig 6C), which also subsequently caused the decreased T-wave amplitude.

**Fig 7:**
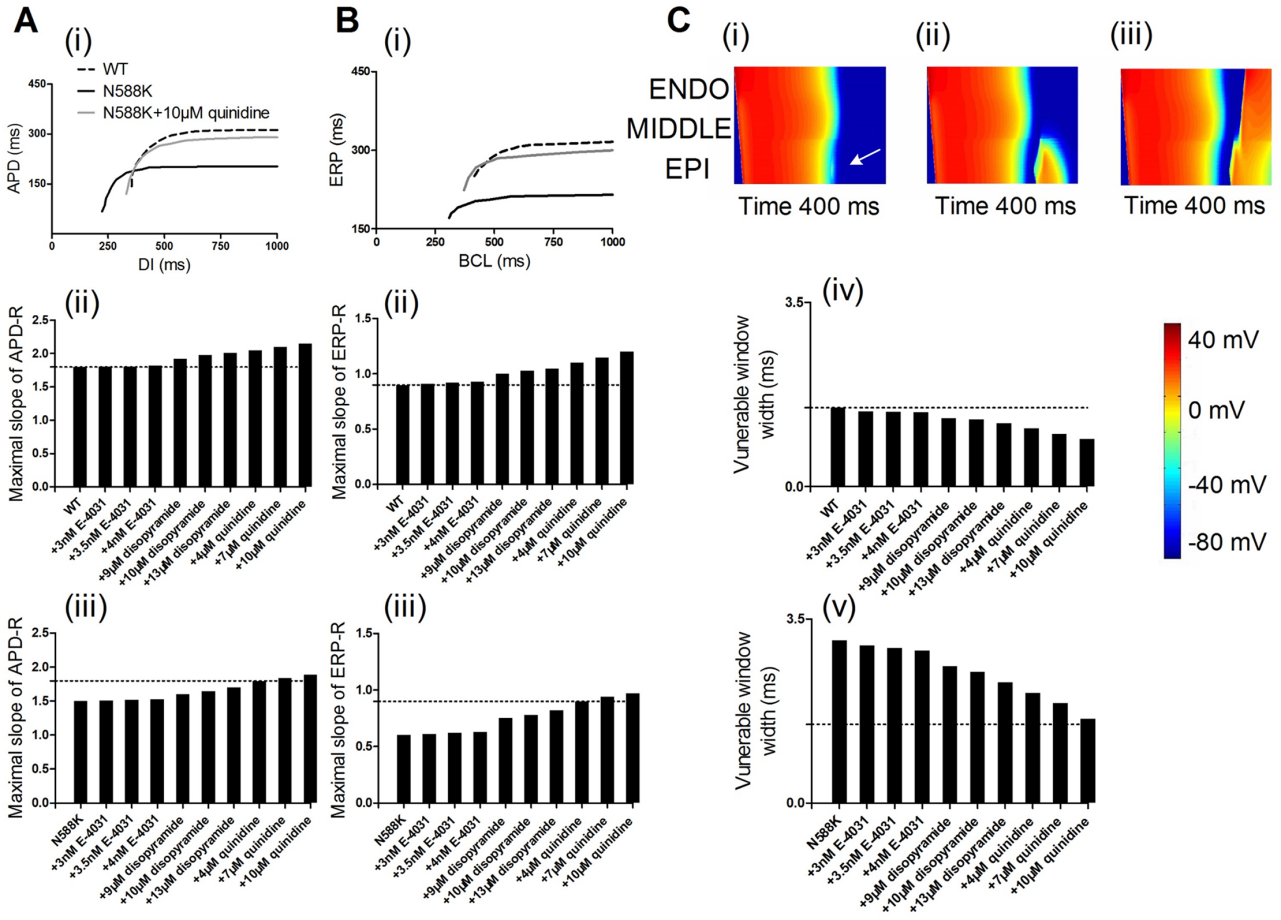
APR-R and ERP-R curve of EPI cells and vulnerable time window along the 1D transmural strand. (A) APD-R curve (i) and maximal slope for in the WT (ii) and N588K (iii) conditions. (B) ERP-R curve (i) and maximal slope for in the WT (ii) and N588K (iii) conditions. (C) Space-time plot of electrical excitation propagation and response of the ventricular tissue to a test stimulus. APs are mapped into a color spectrum ranging from -85 mV to +45 mV. Space runs from top (ENDO) to bottom (EPI). Time runs from left (0 ms) to right (400 ms). Bidirectional block (i). Unidirectional block (ii). Bidirectional conduction (iii). Measured vulnerable time window width of the ventricular tissue in the WT (iv) and N588K (v) conditions.

Simulation of the drug quinidine effects steepened the APD-R curve for the ventricular EPI cell model and caused a rightward shift of the APD-R curve (see Fig 7A). The maximal slopes of the APD-R were increased. These results suggested a regain of rate-adaptation of ventricular APD, indicating anti-arrhythmic effects of quinidine on SQT1 M588K mutation. Simulation of the drug quinidine effects also steepened the ERP-R curve for the ventricular EPI cell model (Fig 7B) and caused a rightward shift of the ERP-R curve with increased maximal slopes. These results also suggested a regain of rate-adaptation of ventricular ERP, also indicating anti-arrhythmic effects of quinidine on SQT1 M588K mutation. The temporal vulnerability (vulnerable time window) of ventricular tissue to unidirectional conduction block was measured in response to a test stimulus. Results are shown in Fig 7C. It was shown that the test stimulus was applied sufficiently early, during and after the vulnerable time window that produced bidirectional conduction block (Fig 7C(i)), unidirectional block (Fig 7C(ii)) and bidirectional conduction (Fig 7C(iii)), respectively. Fig 7C(iv and v) shows the width of the vulnerable time window, during which a test stimulus led to unidirectional conduction block in the WT, N588K, drug-in-action conditions. The measured vulnerable time window decreased from 1.5 ms in the WT (control) condition to 1.43, 1.43, 1.42, 1.3, 1.27, 1.2, 1.1, 1.0 and 0.9 ms in the presence of 3 nM, 3.5 nM and 4 nME-4031, 9 μM, 10 μM and 13 μM disopyramide, and 4, 7 and 10 μM quinidine conditions, respectively, and it decreased from 3.1 ms in the N588K (control) condition to 3.0, 2.95, 2.9, 2.6, 2.5, 2.3, 2.1, 1.9 and 1.6 ms, respectively.

### Effects of E-4031, disopyramide and quinidine on SQT1 in the 2D regular and realistic tissue model

**Fig 8:**
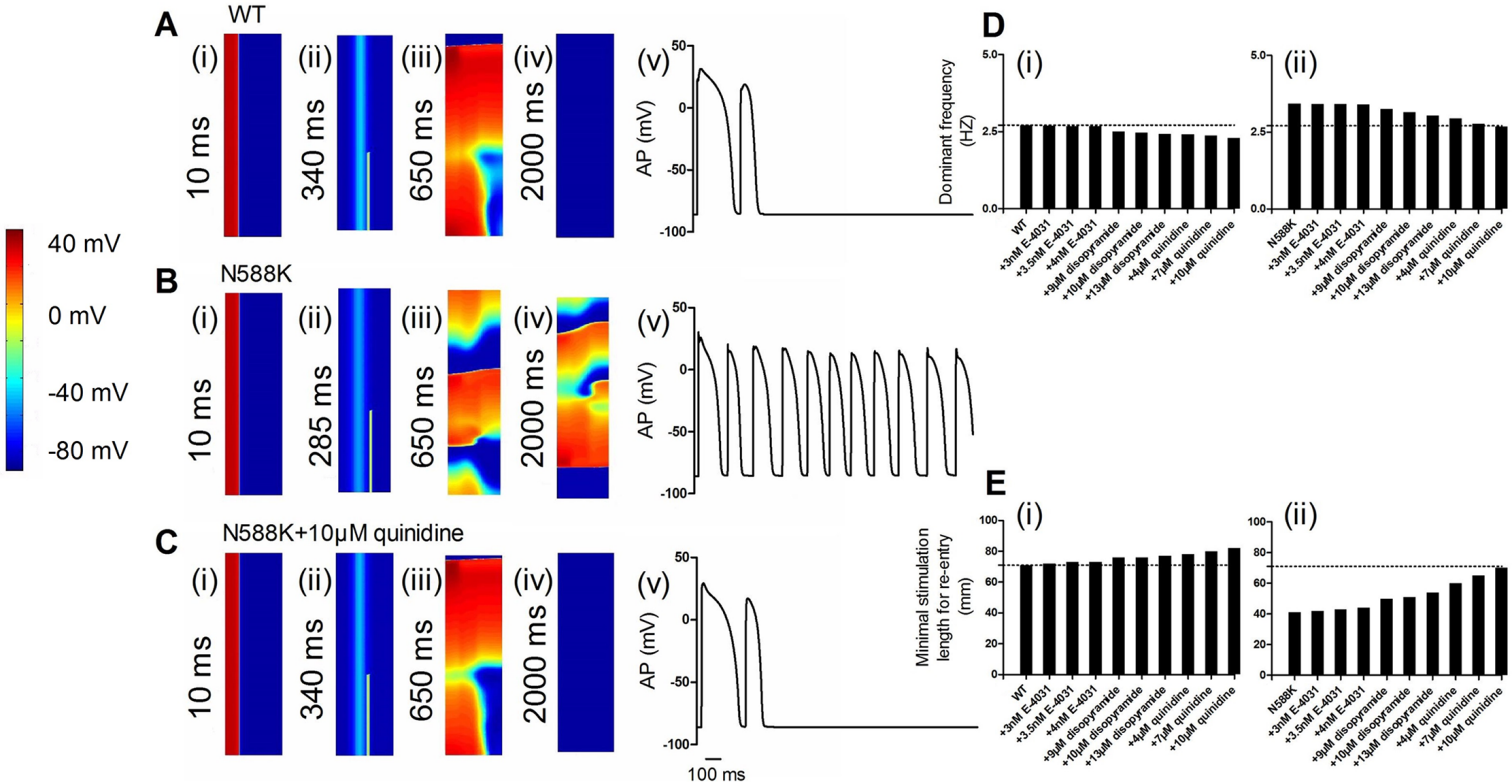
Snapshots of initiation and propagation of re-entry in 2D regular ventricular models. (A) A planar wave generated by S1 at the ENDO end, which propagates through the MIDDLE and then towards the EPI in the WT condition. Snapshots at time = 10 ms (i). The S2 applied to the MIDDLE-EPI junction during the vulnerable time window of the ventricular tissue. Snapshots at time = 340 ms (ii), 650 ms (iii), 2000 (iv) and evolution of the AP of an EPI cell (v). (B) Snapshots of re-entry in the N588K condition at 10 ms (i), 285 ms (ii), 650 ms (iii), 2000 ms (iv) and evolution of the AP of an EPI cell. (C) Snapshots of re-entry in the N588K+10μM quinidine condition at 10 ms (i), 340 ms (ii), 650 ms (iii), 2000 ms (iv) and evolution of the AP of an EPI cell. Quinidine at a dose of 10 μM prevented re-entrant wave in the N588K condition. (D) Computed dominant frequency of electrical activity recorded from the ventricle in WT (i) and N588K (ii) conditions. (E) Measured minimal spatial length of a test stimulus that provides a sufficient substrate for the formulation of a re-entrant circuit in WT (i) and N588K (ii) conditions.

Fig 8 shows the re-entrant excitation waves via pseudo-color plots of cell APs. An S1 stimulus was applied to the ENDO end to evoke an electrical excitation wave that propagated through the MIDDLE and then towards the EPI in the WT (Fig 8A), N588K (Fig 8B) and quinidine-in-action (Fig 8C) conditions. After a time delay (WT: 340 ms; N588K: 285 ms; N588K+ 10 μM quinidine: 340 ms), an S2 was applied to the MIDDLE-EPI junction that produced a unidirectional conduction, forming a re-entrant excitation wave. In the WT condition, the initiated re-entry was unstable, which led to self-termination, as showed in Fig 8A(iv). Fig 8B(iv) shows that the N588K mutation stabilized and sustained re-entry. Quinidine prevented re-entry as shown in Fig 8C(iv). Fig 8A(v), 8B(v) and 8C(v) shows a recording of the evolution of the AP of a cell in the tissue in the WT, N588K, quinidine-in-action conditions, respectively. Fig 8D shows the dominant frequency from 2.71 Hz for WT condition to 2.69, 2.68, 2.67, 2.51, 2.47, 2.43, 2.41, 2.38 and 2.30 Hz in the presence of 3 nM, 3.5 nM, 4 nM E-4031, 9 μM, 10 μM and 13 μM disopyramide, and 4, 7, and 10 μM quinidine conditions, and from 3.43 Hz for N588K condition to 3.42, 3.41, 3.40, 3.25, 3.15, 3.05, 2.95, 2.78 and 2.68 Hz, respectively. We then proceeded to measure the minimum spatial size of the S2 during the vulnerable time window (Fig 8E). The estimated spatial length of the S2 increased from 71 mm in WT condition to 72, 73, 73, 76, 76, 77, 78, 80 and 82 mm in the presence of 3 nM, 3.5 nM, 4 nME-4031, 9 μM, 10 μM, 13 μM disopyramide, 4, 7 and 10 μM quinidine conditions, and increased from 41 mm in N588K condition to 42, 43, 44, 50, 51, 54, 60, 65 and 70 mm, respectively. Prolongation of APD due to the drug action increased the wavelength of the ventricular excitation wave and increased the minimum length of S2 stimulus.

**Fig 9:**
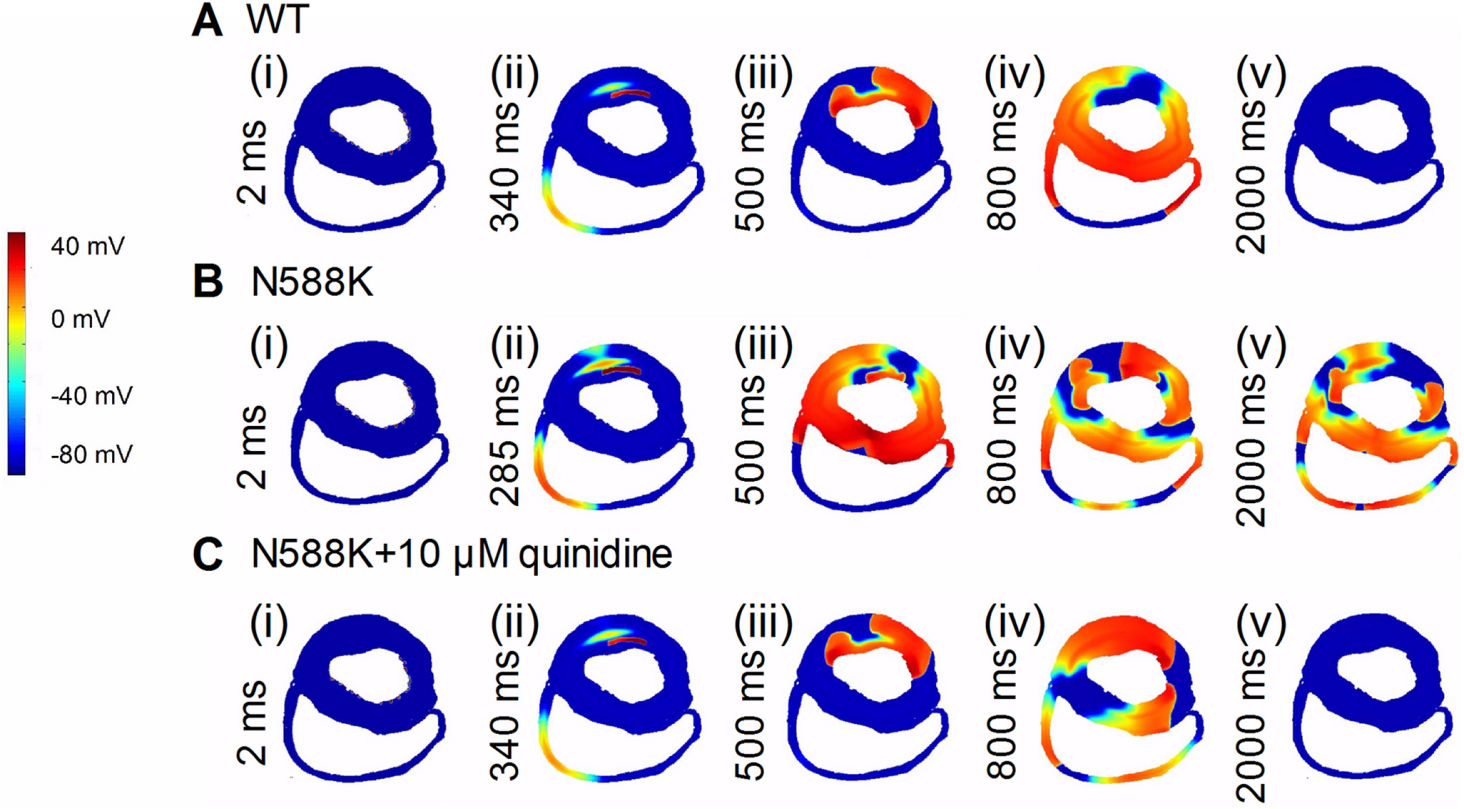
Snapshots of initiation and propagation of re-entry in 2D realistic ventricular models. (A) An excitation wave generated by S1 at the ENDO end in the realistic 2D model cross-section of ventricles in the WT condition. Snapshots at time = 2 ms (i). Application of a test S2 stimulus into the refractory. Snapshots at time = 340 ms (ii). Snapshots of the spiral wave at time = 500 ms (iii), 800 ms (iv) and 2000 ms (v). (B) Snapshots of re-entry in the N588K condition at 2 ms (i), 285 ms (ii), 500 ms (iii), 800 ms (iv) and 2000 ms (v). The induced spiral wave persisted in N588K condition and broke-up forming regenerative multiple re-entrant wavelets. (C) Snapshots of re-entry in the N588K+10μM quinidine condition at 2 ms (i), 340 ms (ii), 500 ms (iii), 800 ms (iv) and 2000 (v). 10μM quinidine terminated the multiple wavelets.

Results of 2D realistic human ventricular tissue are shown in Fig 9. In response to a test S2 stimulus applied in the ENDO region, within the vulnerable time window (WT: 340 ms; N588K: 285 ms; N588K+10 μM quinidine: 340 ms), a re-entry was initiated in the left ventricular wall as shown in Fig 9A(ii) for the WT and Fig 9B(ii) for the N588K mutation and Fig 9C(ii) for the N588K+10 μM quinidine conditions. Subsequent conduction of the induced wave is shown by snapshots induced in Fig 9A(iii-v) for the WT and Fig 9B(iii-v) for the N588K and Fig 9C(iii-v) for the N588K+10μM quinidine conditions. For the WT condition, the initiated re-entrant excitation wave self-terminated (Fig 9A(v)). However, with the N588K mutation, re-entrant excitation wave was sustained (Fig 9B(v)). With the application of 10 μM quinidine in the N588K condition, wavelets were eliminated, and re-entrant excitation wave was terminated by meandering out of tissue border (Fig 9C(v)). Similar results were observed from 2D idealized, regular models. Though the termination was due to re-entry meandering, this is also considered to be antiarrhythmic as there are many non-excitation regions (i. e., connective tissues, opening valves) in the ventricles which can be taken as tissue borders.

## Discussion

### Summary of the major findings

The proband in whom the *KCNH2* N588K mutation was identified had a QT_c_ interval of 288 ms, whereas his son had a QT_c_ of 293 ms. It is of particular note that with the use of quinidine, mimicking the effects on the proband, we found that the simulated QT interval extended from 286 ms in the N588K condition to 382 and 405 ms in the presence of 7 and 10 μM quinidine, which is within the normal range of QT interval between 363 and 421 ms. The findings are summarized as follows: (i) the simulated drugs E-4031 and disopyramide had hardly noticeable effects on APD, whereas quinidine caused a significant prolongation of APD; (ii) quinidine extended the QT interval on the computed pseudo-ECG, and decreased the T-wave amplitude, close to that in the WT condition; (iii) quinidine prolonged ventricular cell APD_90_ across the ventricle wall, and decreased transmural dispersion of APD; (iv) quinidine decreased the maximal transmural AP heterogeneity (*δV*), which contributed to the decreased T-wave amplitude; (v) quinidine decreased the tissue temporal vulnerability to the genesis of uni-directional conduction by a test stimulus; (vi) quinidine increased the minimal substrate size of tissue that required to initiate and maintain re-entrant waves, and prevented and terminated re-entrant waves as shown in both regular and realistic tissue models of the human ventricle; (vii) quinidine exhibited significantly better therapeutic efficacy than E-4031 and disopyramide. These major findings substantiate the causal link between the antiarrhythmic drug quinidine and QT interval prolongation and the decreased T-wave amplitude and, moreover, provide an explanation for decreased susceptibility to re-entry and prevention and termination of re-entrant arrhythmia in SQT1 patients.

### Significance of the Study

Previous studies [12,13] have used a mathematical model of the heart to investigate the pro-arrhythmic effects of *hERG* N588K mutation on ventricular cell APD shortening and characteristics of computed pseudo-ECGs. However, simulations addressing the effects of antiarrhythmic drugs on ventricular cell AP and tissue have not been performed until now. Furthermore, our simulations were able to reproduce the known effects of *hERG* N588K mutation on the electrophysiological activity of the ventricles, which provided the first step towards the validation of the use of the cardiac model in assessing the effects of drugs on SQT1.

Data regarding pharmacological therapy for SQT1 are very limited [52]. An only prior experimental study [17] employing the whole-cell patch clamp, has used five canonical inhibitors including E-4031, amiodarone, quinidine, propafenone and disopyramide, to investigate the link between impaired inactivation and altered drug potency. Nevertheless, it did not examine whether or not these drugs would prolong the QT interval in SQT1 conditions. The present study extends our previous work [13] demonstrating the mechanisms by which the *hERG* N588K mutation facilitates and perpetuates ventricular arrhythmias. Here, we focus on investigating the pharmacology for SQT1 related N588K mutation in *KCNH2*. The different drugs including E-4031, disopyramide and quinidine have been studied. Our results showed that once the drugs were applied, the APD_90_ of each cell was lengthened, QT interval was prolonged and T-wave amplitude was decreased. As expected, the QT interval prolongation is explained by an APD prolongation caused by drugs, whereas the decrease in the T-wave amplitude correlates with a reduced dispersion of repolarization. Previous studies [53–55] have linked changes in T-wave amplitude, which is the gradient of the transmembrane potential in the ventricles. Moreover, the fundamental importance of the maximal transmural AP heterogeneity and APD_90_ dispersion in SQTS has been demonstrated in our previous studies [13,37,46]. To elucidate the effects of E-4031, disopyramide and quinidine on the maximal transmural AP heterogeneity and APD_90_ dispersion, the action of drugs on a ventricular strand model has been studied. In this study, we quantified the ventricular gradient by computing the dispersion of the ventricular APD_90_ across the ventricular wall; we found that drugs caused decreased APD dispersion and the maximal transmural AP heterogeneity. A direct correlation was observed between the changes in APD and the QT interval caused by drugs. These changes can account not only for a prolonged QT interval but can also account for a decreased T-wave amplitude.

Quinidine decreased tissue’s temporal vulnerability for unidirectional conduction block and increased the critical size of the tissue necessary to accommodate re-entrant waves, thus suppressing the initiation and maintenance of re-entrant excitation waves. This result was due to the prolonged APD and ERP and wavelength of excitation waves. Therefore, the re-entrant wave was initiated with greater difficulty, owing to the longer APD and ERP and larger wavelength of the re-entrant excitation wave. In the 2D realistic tissue model, the multiple re-entrant wavelets were terminated in the presence of quinidine, indicating that a transition from fibrillation-like to control (WT) electrical excitation waves.

### Limitations of the study

Limitations of the ten Tusscher *et al*. [27] model for human ventricular myocytes have been discussed in detail elsewhere [13,27,37,46], so that will not be covered again here. Although such a model is not as complete as the newest model [36], it is sufficient for the purpose of this study as the simulated effects of quinidine on SQT1 are in accordance with the clinical phenomenon. In this study, we did not consider the effect of the atria, mechano-electric coupling and feedback, and coronary flow, which might influence the effects of drugs on SQTS.

In this study, the SQT1 data extracted from literature [17] are on homozygous mutant channels (N588K/N588K) versus wild-type channels (WT/WT). Accordingly, simulations were performed on carriers of the homozygous N588K rather than SQT1 patients, who are heterozygous (WT/N588K). Therefore, for future work, integration of heterozygous (WT/N588K) data could provide better insight into the effects of quinidine on SQT1 patients. Another shortcoming is the absence of beta subunits that may interfere with the *I*_Kr_ kinetics, e.g., Mink-related peptide 1 (MiRP1, encoded by the gene *KCNE2*), as demonstrated by McPate *et al*. [28]. Furthermore, there are more experimental data on the N588K mutation than those reported by McPate *et al*. [17], i. e., studies by Brugada *et al*. [5] and Grunnet *et al*. [56]. Consideration of a combination of those N588K mutation data could facilitate more thorough investigations of SQT1.

The action of drugs at the ion channel level was simulated using a simple pore block theory [29]. This method did not incorporate voltage-, state-, frequency-dependent block effects, but it successfully reproduced the required drug-induced decrease in conductance. The use of the simple pore block theory is justified by the match between simulation data and clinical data. In our simulations, the results of quinidine on SQT1 are consistent with the clinical studies (see Discussion section). Regarding the experimental conditions, many factors that can influence the binding of a drug to ion channels are increasingly being modeled, including oxygen, the concentration of ions, temperature and pH [57,58]. However, pharmacological screening does not usually record sufficient data [58]. For conditions with variable pacing rates, conductance block approximation may be insufficient for the simulation of drug actions.

Nevertheless, whilst it is important that potential limitations are made explicit, these limitations do not influence fundamentally the conclusions that can be drawn on likely mechanisms by which quinidine prevents and terminates arrhythmias in SQT1.

## Conclusions

As the SQTS patient is very rare, extensive clinical trials are currently not feasible. However, *in silico* approach can be a powerful tool for assessing drug-induced APD/QT interval prolongation, as well as helping to dissect underlying mechanisms. In this study, we presented a simulation study showing the antiarrhythmic effects of quinidine, disopyramide and E-4031 on SQT1. On the basis of the simulations, it can be concluded that antiarrhythmic drug quinidine causally not only linked to QT interval prolongation but also caused to decreased APD dispersion and maximal transmural AP heterogeneity (*δV*), which contributed to the decreased T-wave amplitude. Additionally, quinidine reduced tissue’s temporal vulnerability to the genesis of re-entry by a test stimulus and increased tissue ERP that prevented and terminated re-entrant excitation waves in the 2D regular and realistic tissues. In conclusion, on one hand, the findings of this study provide an explanation for clinical phenomenon of treating SQT1 patient by quinidine, and demonstrate antiarrhythmic effects of quinidine on SQT1 and suggest quinidine is a potential pharmacological agent for treating SQT1 patients; on the other hand, the ventricular models used in this study may have further utility for probing the pharmacological effects of other antiarrhythmic agents on SQTS.

## Supporting information

S1 Text An appendix showing the *I*_Kr_ Markov chain model equation parameters for WT and N588K conditions. (DOCX)

S1 Fig Pseud-ECGs for the different EPI: MIDDLE: ENDO *I*_Kr_ density ratios of 1.0:1:1, 1.5:1:1, 1.6:1:1 and 1.7:1:1. (A) In WT condition. (B) In N588K condition.
(TIF)

S1 Video WT re-entry in a 2D regular tissue. Initiation and conduction of re-entrant excitation waves in a 2D regular model of the transmural ventricle under the WT condition. A planar wave generated by an S1 stimulus at the ENDO end propagates through MIDDLE and then towards the EPI end. An S2 stimulus is applied to the EPI-MIDDLE junction during the vulnerable time window at 340 ms, which develops into a re-entrant excitation wave. The re-entrant excitation wave self-terminates within 2000 ms.
(MP4)

S2 Video N588K re-entry in a 2D regular tissue. Initiation and conduction of re-entrant excitation waves in a 2D regular model of the transmural ventricle under the N588K condition. A planar wave generated by an S1 stimulus at the ENDO end propagates through MIDDLE and then towards the EPI end. An S2 stimulus is applied to the EPI-MIDDLE junction during the vulnerable time window at 285 ms, which develops into a re-entrant excitation wave. The re-entrant excitation wave persists under the N588K mutant condition.
(MP4)

S3 Video N588K+quinidine re-entry in a 2D regular tissue. Initiation and conduction of re-entrant excitation waves in a 2D regular model of the transmural ventricle under the WT condition. A planar wave generated by an S1 stimulus at the ENDO end propagates through MIDDLE and then towards the EPI end. An S2 stimulus is applied to the EPI-MIDDLE junction during the vulnerable time window at 340 ms, which develops into a re-entrant excitation wave. The re-entrant excitation wave self-terminates within 2000 ms.
(MP4)

S4 Video WT re-entry in a 2D realistic tissue. Re-entrant excitation wave generated by a test S2 stimulus during the vulnerable time window at 340 ms. The initiated re-entrant excitation wave self-terminates within 2000 ms.
(MP4)

S5 Video N588K re-entry in a 2D realistic tissue. Re-entrant excitation wave generated by a test S2 stimulus during the vulnerable time window at 285 ms. The initiated re-entrant excitation wave transition from transmural re-entrant wave with tip rotating within the ventricle wall to anatomical re-entrant wave with tip rotating around the ventricle boundary. The re-entrant excitation wave persists under the N588K condition and breaks up forming multiple re-entrant wavelets.
(MP4)

S6 Video N588K+quinidine re-entry in a 2D realistic tissue. Re-entrant excitation wave generated by a test S2 stimulus during the vulnerable time window at 340 ms. With the application of quinidine in the N588K condition, the initiated re-entrant excitation wave was terminated within 2000 ms.
(MP4)
