# Supplementary material for: In silico assessment of the effects of quinidine, disopyramide and E-4031 on short QT syndrome variant 1 in the human ventricles
Source: PLoS One. 2017 Jun 20;12(6):e0179515. doi: 10.1371/journal.pone.0179515 (PMC5478111; doi:10.1371/journal.pone.0179515)
Supplement: S1 Text — (DOCX) [file pone.0179515.s001.docx]

# *In Silico* Assessment of the Effects of Quinidine, Disopyramide and E-4031 on Short QT Syndrome Variant 1 in the Human Ventricles

Cunjin Luo^1^, Kuanquan Wang^1*^, Henggui Zhang^1,2,3*^

^1^ School of Computer Science and Technology, Harbin Institute of Technology (HIT), Harbin, China

^2^ School of Physics and Astronomy, The University of Manchester, Manchester, United Kingdom

^3^ Space Institute of Southern China, Shenzhen, China

*I*_Kr_ model rate transition equations [[1](#_ENREF_1)]:

WT:

$$\alpha_{1}=2.172$$

$$\beta_{1}=1.077$$

$$\alpha_{2}=0.00655\times e^{0.027735765\times\left(V-36\right)}$$

$$\beta_{2}=0.001908205\times e^{-0.0148902V}$$

$$\alpha_{i}=0.04829\times e^{-0.039984\times\left(V+25\right)}\times\left(\frac{4.5}{K_{O}}\right)$$

$$\beta_{i}=0.2624\times e^{\left(0.000942V\right)}\times\left(\frac{4.5}{K_{O}}\right)^{0.3}$$

$$\alpha=0.00555\times e^{0.05547153\left(V-12\right)}$$

$$\beta=0.002357\times e^{-0.036588V}$$

$$\mu=\frac{\alpha_{i}\beta_{2}}{\beta_{i}}$$

N588K:

$\alpha_{1}=2.172$ $\beta_{1}=0.5385$

$$\alpha_{2}=0.001965\times e^{0.05547153\times\left(V-21\right)}$$

$$\beta_{2}=2.260489\times10^{-6}\times e^{-0.0925782V}$$

$$\alpha_{i}=0.439\times e^{-0.02352\times\left(V+40\right)}\times\left(\frac{4.5}{K_{O}}\right)$$

$$\beta_{i}=0.0164\times e^{0.000942\times\left(V+15\right)}\times\left(\frac{4.5}{K_{O}}\right)^{0.3}$$

$$\alpha=0.00555\times e^{0.05547153\left(V+3\right)}$$

$$\beta=0.002357\times e^{-0.036588V}$$

$$\mu=\frac{\alpha_{i}\beta_{2}}{\beta_{i}}$$

1. Adeniran I, McPate MJ, Witchel HJ, Hancox JC, Zhang H (2011) Increased vulnerability of human ventricle to re-entrant excitation in hERG-linked variant 1 short QT syndrome. PLoS Comput Biol 7: e1002313.
