# Supplementary figures and images for: In silico assessment of the effects of quinidine, disopyramide and E-4031 on short QT syndrome variant 1 in the human ventricles
Source: PLoS One. 2017 Jun 20;12(6):e0179515. doi: 10.1371/journal.pone.0179515 (PMC5478111; doi:10.1371/journal.pone.0179515)

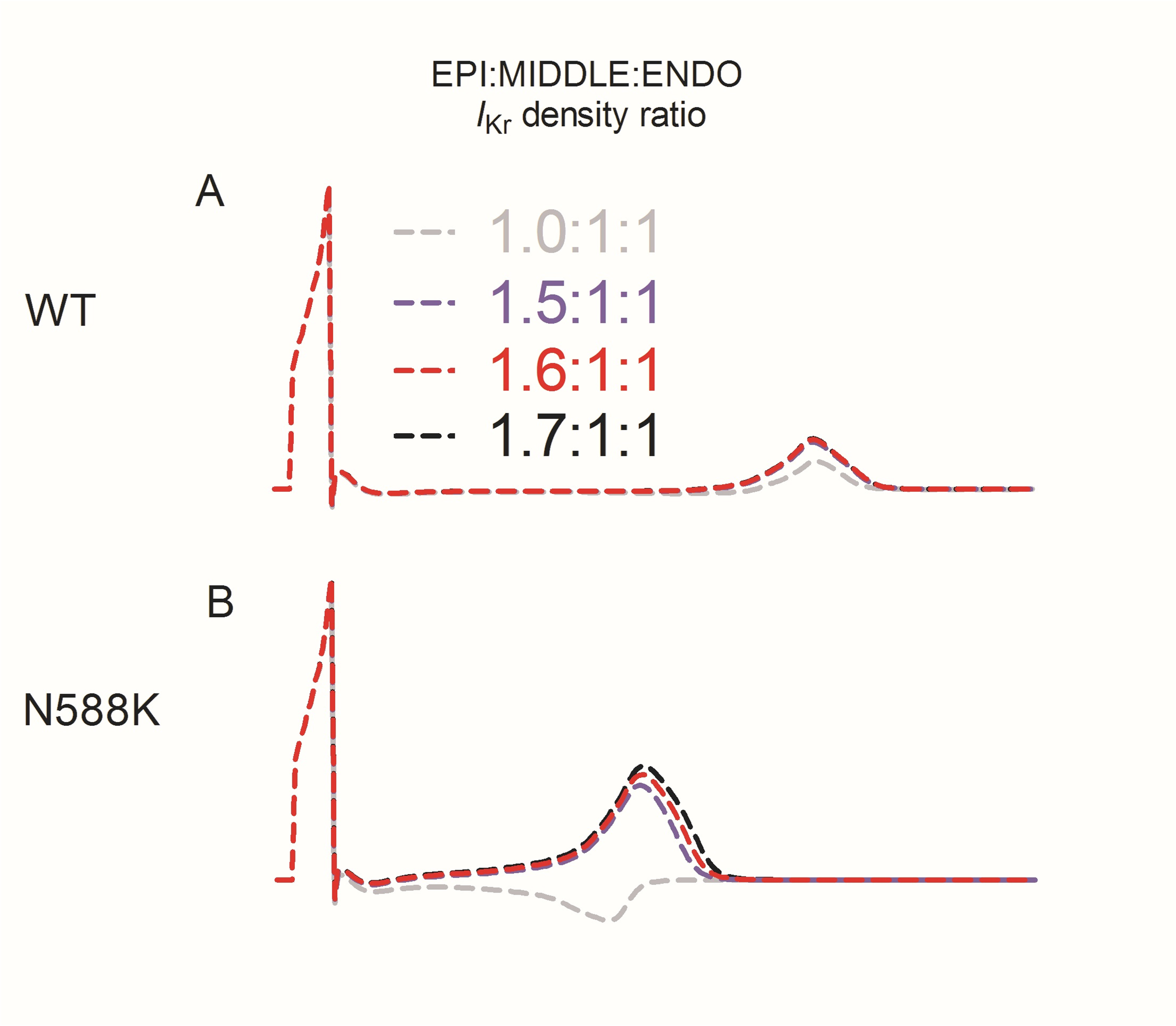

Supplement: S1 Fig — (A) In WT condition. (B) In N588K condition. (TIF) [file pone.0179515.s002.tif]
